# 134. Characterization of circulating clinical multi-drug resistant and methicillin resistant Staphylococcus aureus isolates in Peru

**DOI:** 10.1093/ofid/ofac492.212

**Published:** 2022-12-15

**Authors:** Jesús D Rojas, Enrique A Canal, Manuela M Bernal, Rhonda A Lizewski, Paul A Rios

**Affiliations:** Vysnova Partners Inc., Reston, Virginia; Naval Medical Research Unit-6, Callao, Callao, Peru; Naval Medical Research Unit-6, Callao, Callao, Peru; Naval Medical Research Unit-6, Callao, Callao, Peru; Naval Medical Research Unit-6, Callao, Callao, Peru

## Abstract

**Background:**

Multi-drug resistant and methicillin resistant *Staphylococcus aureus* (MDR-SA and MRSA, respectively) constitute a significant threat to hospitalized patients. Surveillance for antibiotic resistance is needed in settings where over-the-counter antibiotic use is widespread to identify increasing antibiotic resistance that limits treatment options of nosocomial infections.

**Methods:**

We performed phenotypic and genotypic characterizations of MDR-SA prospectively obtained from hospitals in Lima, Peru between 2015 - 2018. We collected 3103 bacterial isolates from clinical specimens. Bacterial samples were identified by conventional microbiology and BD Phoenix, and antimicrobial susceptibility testing (AST) was performed by disc diffusion, E-test and BD Phoenix. Additionally, multiplex PCR detected *mecA*, *femA*, and *lukS* in MDR-SA isolates. We selected 80 *S*. *aureus* isolates that were resistant to ≥ 3 antimicrobial classes and/or resistant to oxacillin or cefoxitin for genomic characterization using an Illumina NextSeq 500 sequencer.

**Results:**

Testing identified 232/3103 isolates as *S*. *aureus*, and 80/232 (34%) isolates were MDR-SA, resistant to up to nine different antimicrobial classes. We observed high proportions of resistance to aminopenicillins 80/80 (100%), macrolides 71/80 (89%), lincomycins 69/80 (86%), quinolones 66/80 (83%), aminoglycosides 38/46 (82%). Notably, 75/80 (94%) isolates were MRSA. Interestingly, AST revealed that 81% had inducible resistance to clindamycin. In contrast, all isolates were susceptible to vancomycin, linezolid, and daptomycin. Multiplex PCR revealed that 5% of isolates carried the *lukS* gene and the *mecA* gene. 76 % of the isolates carried a bifunctional aminoglycoside modifying enzyme and a aminoglycoside O-nucleotidyltransferase enzymes that confer resistance to aminoglycosides. We detected the *erm*A gene in 79% of the isolates. *In silico* multilocus sequence typing (MLST) assigned the sequence type 5 to 91.2% MRSA isolates with multiple high related clusters observed, indicating the possibility of nosocomial dissemination and possible outbreak events.

**Conclusion:**

Antibiotic pressure on the resistance patterns of *S. aureus* is evident in the high levels of resistance seen in these clinical isolates.

**Disclosures:**

**All Authors**: No reported disclosures.

